# Proteomic Signatures of the Desmoplastic Invasion Front Reveal Collagen Type XII as a Marker of Myofibroblastic Differentiation During Colorectal Cancer Metastasis

**DOI:** 10.18632/oncotarget.451

**Published:** 2012-03-08

**Authors:** George S. Karagiannis, Constantina Petraki, Ioannis Prassas, Punit Saraon, Natasha Musrap, Apostolos Dimitromanolakis, Eleftherios P. Diamandis

**Affiliations:** ^1^ Department of Laboratory Medicine and Pathobiology, University of Toronto, Toronto, Ontario, Canada; ^2^ Department of Pathology and Laboratory Medicine, Mount Sinai Hospital, Toronto, Ontario, Canada; ^3^ Pathology Department, Metropolitan Hospital, Athens, Greece; ^4^ Department of Clinical Biochemistry, University Health Network, Toronto, Ontario, Canada

**Keywords:** Colorectal cancer, Cancer-associated fibroblasts, Desmoplasia, Proteomics, Secretome, Collagen type XII

## Abstract

Cancer-associated fibroblasts (CAFs), represent a pivotal compartment of solid cancers (desmoplasia), and are causatively implicated in cancer development and progression. CAFs are recruited by growth factors secreted by cancer cells and they present a myofibroblastic phenotype, similar to the one obtained by resident fibroblasts during wound healing. Paracrine signaling between cancer cells and CAFs results in a unique protein expression profile in areas of desmoplastic reaction, which is speculated to drive metastasis. In an attempt to decipher large-scale proteomic profiles of the cancer invasive margins, we developed an *in vitro* coculture model system, based on tumor-host cell interactions between colon cancer cells and CAFs. Proteomic analysis of conditioned media derived from these cocultures coupled to mass spectrometry and bioinformatic analysis was performed to uncover myofibroblastic signatures of the cancer invasion front. Our analysis resulted in the identification and generation of a desmoplastic protein dataset (DPD), consisting of 152 candidate proteins of desmoplasia. By using monoculture exclusion datasets, a secretome algorithm and gene-expression meta-analysis in DPD, we specified a 22-protein “myofibroblastic signature” with putative importance in the regulation of colorectal cancer metastasis. Of these proteins, we investigated collagen type XII by immunohistochemistry, a fibril-associated collagen with interrupted triple helices (FACIT), whose expression has not been reported in desmoplastic lesions in any type of cancer. Collagen type XII was highly expressed in desmoplastic stroma by and around alpha-smooth muscle actin (α-SMA) positive CAFs, as well as in cancer cells lining the invasion front, in a small cohort of colon cancer patients. Other stromal markers, such as collagen type III, were also expressed in stromal collagen, but not in cancer cells. In a complementary fashion, gene expression meta-analysis revealed that COL12A1 is also an upregulated gene in colorectal cancer. Our proteomic analysis identified previously documented markers of tumor invasion fronts and our DPD could serve as a pool for future investigation of the tumor microenvironment. Collagen type XII is a novel candidate marker of myofibroblasts, and/or cancer cells undergoing dedifferentiation.

## INTRODUCTION

The “cancer/tumor invasion front” represents a spatially-organized tumor site, where many aspects of cancer development and progression are regulated at the single- and collective-cell levels. The cancer invasion front is, in fact, the invasive margins of any primary neoplasm that has breached the basement membrane at an earlier point, and is currently excavating a metastatic pathway through the normal underlying stroma, towards the lymphatic or blood vessels [1, 2]. Paracrine signaling between cancer cells and associated stromal cells is almost exclusively present at the invasive sites. Cancer cells tend to deploy a cell-biological program, called epithelial-to-mesenchymal transition (EMT) [3, 4], during which, they lose epithelial shape, polarity and cytoskeletal organization and gain mesenchymal-like properties. These changes are followed by large-scale alterations in their gene expression machinery [3, 5]. EMT, has been considered for a long time as an important trait of metastasizing cells [4]. Cancer cells undergoing EMT detach from the main cancerous core, and obtain a highly-malignant phenotype; this cancer cell subpopulation has been described as “tumor bud” and is now histopathologically evaluated and linked to poor prognosis and patient survival [6].

The cancer-associated stroma is a highly important constituent of the cancer invasion front, in many cases consisting of cancer-associated fibroblasts (CAFs). The accumulation of CAFs peritumorally represents the host response to the presence of neoplastic lesions within the stroma. CAFs are normal host cells, particularly derived by normal quiescent fibroblasts (NFs), although other sources have been also reported (e.g. mesenchymal stem cells, bone marrow-derived stem cells, endothelial cells), which have deployed a specific cell-biological program, termed “myofibroblastic differentiation” [7, 8]. These peritumoral myofibroblasts are characterized by altered proliferative, migratory and contractile behavior compared to NFs. In addition, they fully deploy smooth-muscle-like gene and protein expression machinery, which has been initially noticed in stromal responses in tissue wounding [9]. For instance, CAFs initiate the expression of alpha-smooth muscle actin (α-SMA), which is speculated to provide smooth muscle-like contractile activity, and is the most significant hallmark to distinguish CAFs from NFs [10].

It is now clear that CAFs constitute a rich source of matrix remodeling molecules (e.g. tenascin-C, matrix metalloproteinases) that directly or indirectly affect the metastatic cascade and are responsible for the increased deposition of extracellular matrix (ECM) components at the tumor invasion front, a type of lesion that has been described as “desmoplasia” [7, 9, 11-14]. In addition, CAFs produce multiple growth factors [e.g. insulin growth factor (IGF), hepatocyte growth factor (HGF)], which promote tumor cell proliferation, survival and motility/migration [10, 15]. Moreover, CAFs participate in yet other paracrine signaling pathways that together regulate and promote inflammation [e.g. interleukin-1β (IL-1β), tumor necrosis factor-α (TNF-α)] [16], and angiogenesis [e.g. vascular endothelial growth factor (VEGF)] [17], which further qualifies them as central mediators of various aspects of carcinogenesis. Overall, CAFs seem to serve as a pivotal source of strategies, deployed at both primary and metastatic tumor sites, mediating and supporting the malignant properties of cancer spreading [18, 19].

Currently, high-throughput studies, mostly genome-wide- and proteome-based, are deployed for the scrutinization and subsequent investigation of the tumor microenvironment [20-22]. Emerging novel mass spectrometry-based proteomic strategies have allowed for simultaneous identification and quantification of thousands of proteins, even in complex biological mixtures [23]. Hence, proteomic platforms have been widely developed to provide insight, especially into the rationalized biomarker discovery field, and to additionally provide more systematic understanding of various physiological and pathophysiological, cellular processes [24, 25]. In this study, we attempted to decipher and characterize molecular events of tumor-host cell interactions that occur early in the course of metastasis, using the colorectal cancer desmoplastic microenvironment as a model system. In this approach, we generated *in vitro* cell-contact cocultures of colorectal cancer (CRC) cell lines and colonic NFs, in an attempt to mimic the paracrine signaling milieu of colorectal cancer tumor invasion fronts. Following a previously-described proteomic strategy [24, 26], we subjected these cocultures to comprehensive secretome analysis using liquid chromatography-tandem mass spectrometry (LC-MS/MS), and generated a pool of potential candidates that could be liberated at the tumor invasion front and regulate various aspects of cancer metastasis. This strategy successfully integrates an *in vitro* coculture model system with proteomics, bioinformatics and tissue wide-based expressional studies.

## RESULTS

### Development of a desmoplastic coculture model system

**Figure 1:**
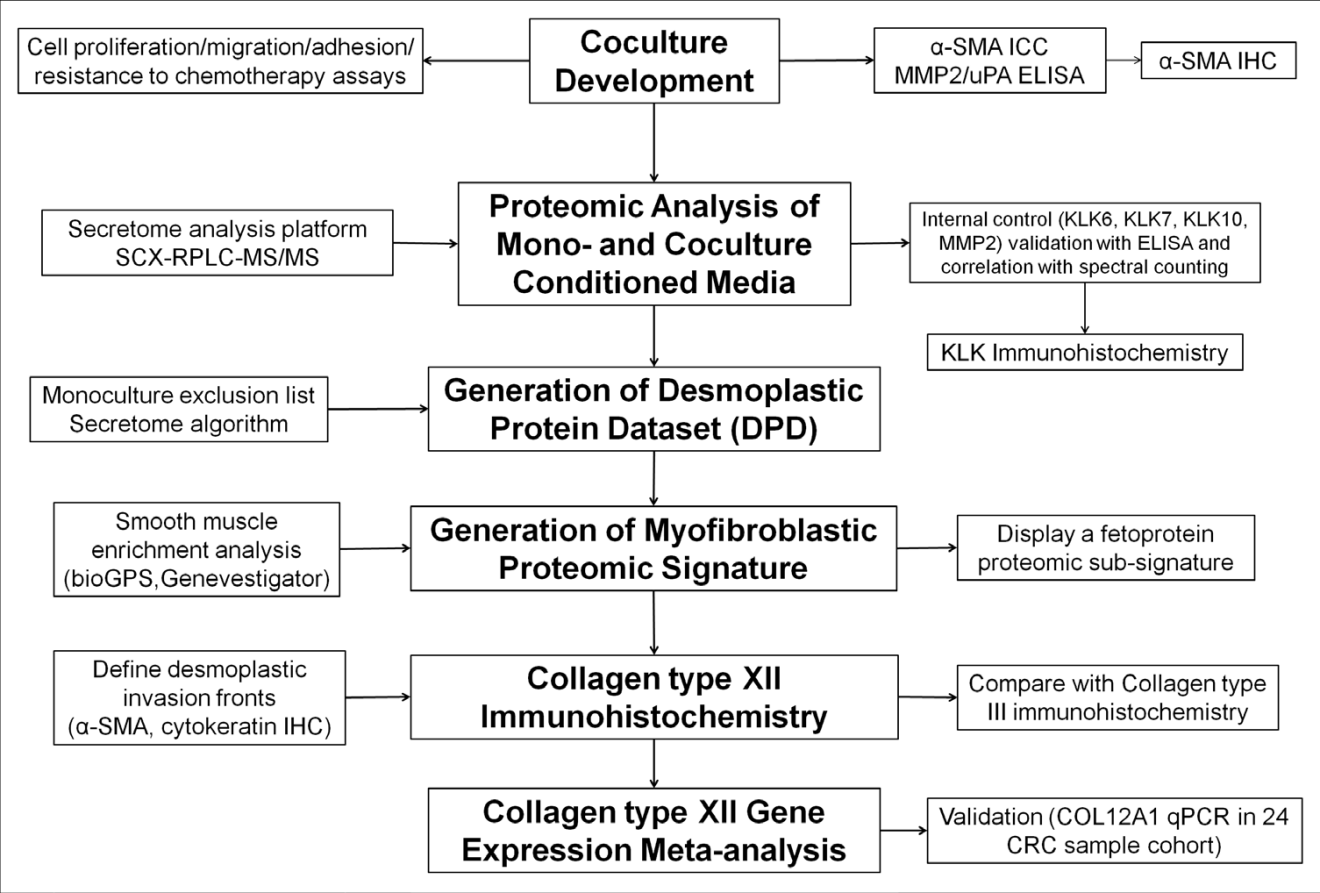
Experimental outline for identification, characterization and validation of desmoplastic proteomic signatures in CRC Nodes in bold represent the experimental steps, while the remaining nodes describe strategies and tools. See also non-standard abbreviations for abbreviated terms.

We tested our strategy (Figure 1), with a “colorectal cancer-colonic fibroblast” tumor-host cell interaction model system, for the following reasons: (A) The contribution of CAFs in the early course of most solid cancers is now well-recognized [19]. (B) Desmoplasia has been thoroughly investigated and associated with progression of gastrointestinal cancers, especially in pancreatic and colorectal cancers [27, 28]. (C) A normal colonic fibroblast cell line (18Co) was commercially available and these cells could be cultured and co-cultured very easily, compared to other types of stromal cells (e.g. endothelial cells, immune cells). Thus, we established *in vitro*, cell-contact colorectal cancer cocultures (Supplementary Figure 1).

Proteomic analysis in only one coculture would probably reveal certain cell line selection biases; by selecting at least two cancer cell lines, we would be able to cross-examine any potential candidates of tumor development and progression, via proteomic delineation of their secretomes. Among the plethora of colon cancer cell lines, we selected the SW480/SW620 as a reasonable *in vitro* system for the screening; this system would allow us to capture some colon cancer heterogeneity, since SW480/SW620 cell lines were obtained from the same patient, but at a different tumor stage [29]. We, therefore, developed cell contact, two-dimensional cocultures of SW480/SW620 cells and 18Co normal colonic fibroblasts (named SW480Co and SW620Co, respectively), and used the relevant monocultures as controls (Supplementary Figure 1). Similar viable cocultures (HT29Co, HCT116Co) were generated with other cancer cell lines (HT29 and HCT116, respectively) (Supplementary Figure 1).

**Figure 2:**
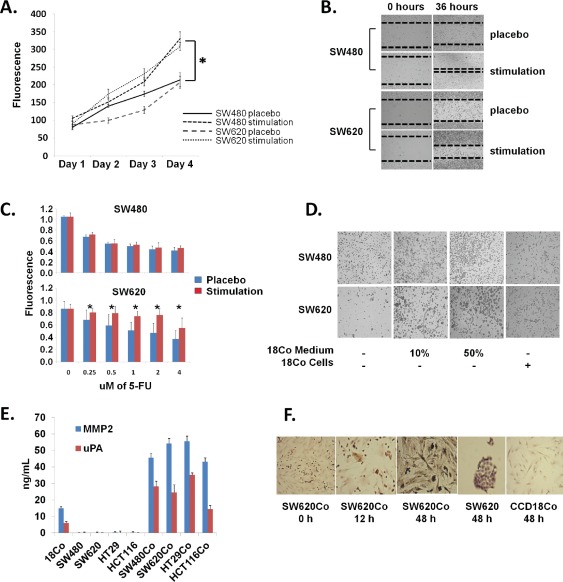
Coculture development (A) Cell proliferation assay (Alamar Blue). SW480 and SW620 colon cancer cell lines were treated with 18Co fibroblast CM or left untreated (placebo) and cell proliferation was measured in a time-dependent manner. Note that 18Co CM-stimulated cancer cells proliferate faster than placebo-treated ones. * *denotes statistical significance* (*p<0.05, t-test*). (B) Cell migration (scratch) assay. SW480 and SW620 colon cancer cell lines were seeded in monolayer, scratched and treated with 18Co fibroblast CM or left untreated (placebo). Note that 18Co CM-stimulated cancer cells heal the *in-vitro* wound faster. (C) Resistance to chemotherapy assay. SW480 and SW620 colon cancer cell lines were treated with 18Co fibroblast CM or left untreated (placebo), following treatment with increasing doses of 5-FU. Note that 18Co CM caused pharmacological rescue in 5-FU-treated SW620 cells, while no significant effect was shown for SW480 cells. * *denotes statistical significance* (*p<0.05, t-test*). (D) Cell adhesion assay. SW480 and SW620 cells were suspended in serum-free CM and plated either in CDCHO, or treated with increasing doses of 18Co fibroblast CM, or on top of pre-seeded fibroblasts, and adhesion was evaluated with microscopy. Note that cancer cells form spheroids and loosely attach to the plates only with preseeded 18Co cells or treated with 18Co CM. (E) MMP2 and uPA ELISA in CM from mono- or co-cultures. Note increased secretion of both proteins in cocultures. (F) α-SMA ICC. Note that, in contrast to monocultures, 18Co cells in coculture conditions express α-SMA. Cancer cells are negative for α-SMA. *All magnifications x10, except third and fifth figure (x40).*

First, we tested whether paracrine signaling between these colon cancer cell lines and 18Co normal colonic fibroblasts could occur, under various *in vitro* coculture setups. To verify this, we collected CM from 2-day monocultures of 18Co NFs and stimulated the SW480/SW620 cells, to investigate whether they could utilize paracrine factors derived from stromal cells. Both SW480 and SW620 colon cancer cell lines displayed statistically significant increases in their growth rate (p<0.05), in a time-dependent cell proliferation assay, when treated with 18Co CM (Figure 2A). This observation was in concordance with cell scratch assays; SW480 and SW620 cells treated with 18Co CM were able to heal the *in vitro* wound faster than the placebo-treated cells, an effect that points to enhanced regulation of cell proliferation and perhaps migration (Figure 2B). Next, we performed an *in vitro* resistance-to-chemotherapy assay, measuring cell viability with the Alamar Blue assay. In this assay, when SW480 cells were treated with 5-FU, a well-known drug used in the FOLFOX adjuvant chemotherapy for colorectal cancer treatment [30], no pharmacological rescue was noticed with the parallel administration of 18Co CM (p>0.05). However, 18Co CM caused a significant rescue in the 5-FU-treated SW620 cells, in a dose-dependent manner (p<0.05) (Figure 2C). This could potentially suggest that SW620 cells might utilize survival factors present in the 18Co CM. We then, performed an *in vitro* cell adhesion assay. In this setup, SW480 and SW620 cells were resuspended in serum-free medium and were subsequently seeded in tissue culture plates. The absence of serum proteins from the CM did not allow these cells to adhere to the plates two days after the initiation of the assay (Figure 2D). However, when these cells were supplemented with increasing doses of 18Co CM (10% or 50%), they formed spheroids with loose attachment on the plates, in a dose-dependent manner (Figure 2D). In concordance with this, when SW480 or SW620 cells were seeded on top of 10% confluent pre-seeded 18Co cells, without stimulation by 18Co CM, some cells managed to adhere onto or away from the fibroblasts (Figure 2D). Overall, these phenotypic observations support the notion that SW480/SW620 cells are capable of utilizing paracrine signals from stromal cells and stimulate various biological processes, such as growth, migration, adhesion and survival.

Before subjecting CM to proteomic analyses, we also wished to test whether paracrine signaling between cancer cells and 18Co NFs could lead to the transdifferentiation of the latter cells into CAFs, an observation that would be in agreement with the literature [8], and would render our rationale more concrete. To evaluate such processes, we measured MMP2 and uPA, two prominent markers of CAFs [9], in mono- and coculture CM, using specific immunoassays. These two factors were not secreted by any of the two colon cancer cell lines, but low levels were detected in the 18Co monocultures (Figure 2E). However, their secretion was significantly increased (p<0.05) in both SW480Co and SW620Co cocultures (Figure 2E). The exact origin of MMP2 and uPA in the cocultures was not defined. These could be secreted by CAFs, or the cancer cells in response to CAF signals [for instance, cancer cells undergoing EMT [6]]. Literature suggests that these factors are stromatogenic, and are most probably secreted by CAFs [9], although other stromal cells (e.g. tumor-associated macrophages) have also been reported to secrete them [31]. These factors were also secreted by our two decoy cocultures HT29Co and HCT116Co but not by either HT29 or HCT116 monocultures (Figure 2E).

We additionally tested the expression of α-SMA, an intermediate filament protein that is known to be expressed by smooth muscle and other smooth muscle-like cells (i.e. myofibroblasts), but not reported to be expressed by NFs [7, 9]. Although some groups have reported that *in vitro* cultured NFs might express small amounts of α-SMA [32, 33], we found that α-SMA expression was higher in cocultured fibroblasts compared to the monocultured ones, while cancer cells did not express it at all, using ICC (Figure 2F). Together, these phenotypic observations demonstrate that *in vitro* interactions of cancer cells with fibroblasts, may lead to the differential expression of prominent markers of CAFs. Thus, our proposed SW480Co/SW620Co coculture model could provide an *in vitro* representation of the tumor invasion front.

### Comprehensive proteomic analysis of mono- and coculture CM

**Figure 3:**
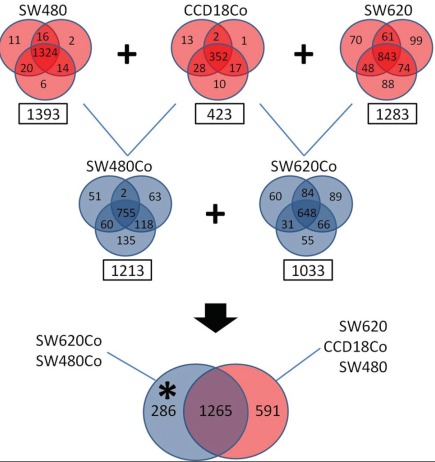
Secretome analysis Proteomic analysis of mono-culture and coculture CM. Venn diagrams show reproducibility among triplicates. In squares are the numbers of identified proteins with a minimum of two peptide hits. Monocultures are depicted with red and cocultures with blue color. The monoculture datasets have been used as exclusion lists to the coculture ones, identifying a dataset of 286 coculture-specific proteins (depicted with an asterisk).

We collected CM from all mono- and cocultures, after a 2-day incubation period and subjected them to comprehensive secretome analysis, following an *in-house* protocol [26], with slight modifications (as explained in Methods). We used the stringent criterion of a minimum of two peptide hits for protein identification and all experiments were performed in triplicate, to increase the accuracy of identification. Therefore, a total of 15 CM samples, corresponding to five experimental conditions (SW480, SW620, 18Co, SW480Co and SW620Co) were collected, trypsin-digested and subjected to SCX-RPLC-MS/MS. We identified 1393, 1213, 423, 1283 and 1033 proteins for SW480, SW620, 18Co, SW480Co and SW620Co, respectively (Supplementary Tables 1-5), with acceptable reproducibility among the replicates (ranging from 63% to 93% in each mono- or coculture) (Figure 3). A total of 2142 non-redundant proteins were identified with the combination of all protein datasets. The complete lists of proteins identified (≥ 2 peptides), including number of unique peptides, IPI accession number, number of assigned spectra and % sequence coverage is presented in Supplemental Tables 1-5 for each experimental condition. Currently, there is no other proteomic study, delineating the secretome of 18Co normal colonic fibroblasts and consequently of the relevant cocultures. However, in previous studies, the secretomes of SW480 and SW620 cell lines have been deciphered through various proteomic approaches [34-36]. Identification of proteins in a comparative context was made in only one of these studies [34], in which, an LC-MS/MS approach was also used. In that study, 796 and 828 proteins were identified for SW480 and SW620 cell lines respectively, with the same criterion of two peptide hits for protein identification. Our secretomes for these two colon cancer cell lines (1293 and 1213 proteins for SW480 and SW620 cell lines, respectively) are more complete.

To test the accuracy of the MS data, we considered a small panel of four secreted internal control proteins, including KLK6, KLK7, KLK10 and MMP2 and investigated their expression through specific immunoassays in all CM subjected to secretome analysis, and compared their levels to the respective spectral counts (independent MS/MS events) from the LC-MS/MS analysis (Supplementary Figure 2). CM from all the mono- and cocultures were collected at days 2 and 4 and were normalized for total protein content, before ELISA analysis. All internal control proteins showed an increase between day 2 and 4 in the cultures, where they were found to be secreted. Specifically, KLK6 was found in all but the 18Co secretome and was identified with more than 10 spectral counts in all replicates of SW480, SW620 and their cocultures. KLK7 was identified with 3 spectral counts in the SW480 secretome; no positive identification was made in SW480Co secretome. Accordingly, KLK7 immunoassay verified this result, as KLK7 was detectable in low amounts (<2.5 ug/L), only in the SW480 supernatant. KLK10 was measured in SW480 and SW480Co secretomes using the specific immunoassay and was identified with more than 7 spectral counts in all replicates in the LC-MS/MS analysis. Finally, MMP2 was secreted in large amounts by 18Co normal fibroblasts (~40 ug/L at day 4), and the relevant cocultures. MMP2 was identified with a large number of spectral counts (~25-50) in all 18Co-containing mono- and cocultures. Thus, our mass spectrometry and immunoassay data suggest that KLKs are probably secreted by cancer cells, while MMP2 is mainly stromatogenic. We also demonstrated *in vivo* relevance of our internal control expression data, using immunohistochemistry. KLK6, KLK7 and KLK10 immunoexpression was observed in the columnar cells of normal colon mucosa. Goblet cells also showed cytoplasmic staining, while most mucin droplets remained unstained. Colorectal adenocarcinoma cases showed a variable cytoplasmic KLK immunoexpression, from negative to strongly positive (Supplementary Figure 3). Increased MMP2 expression in cancer-associated stroma and desmoplasia has been widely demonstrated by other groups [37, 38].

### Generation of a comprehensive “Desmoplastic Protein Dataset” (DPD)

Combination of the two coculture datasets (SW480Co and SW620Co) gave rise to 1551 non-redundant proteins (Figure 3). In an attempt to generate a comprehensive heterotypic dataset of secreted candidate proteins present in desmoplastic invasion fronts, we first used all monoculture datasets (SW480, SW620, 18Co) as exclusion datasets, to retain the “coculture-specific” proteins. This filter allowed us to generate a list of 286 proteins (Figure 3A, Supplementary Table 6), which were either *de novo* expressed in the cocultures, or could be expressed in monocultures in small amounts, not detectable via LC-MS/MS. This filter also gave rise to 591 monoculture-specific proteins and 1265 proteins, present in at least one mono- and one coculture dataset, as shown in the Venn diagrams (Figure 3). Although these 591 monoculture-specific proteins could also be of functional importance, we decided to focus on the 286 protein dataset, since the latter proteins could be potentially present at high concentrations at the cancer invasion front. We cannot exclude the possibility that some of these proteins (probably of low-abundance) were not detected in some monoculture CM, due to methodological and instrumental limitations (relatively low sensitivity). The three biological replicates and the 2-peptide hit criterion should minimize such biases.

Secondly, we filtered the 286 protein dataset through the secretome algorithm (see Methods), to remove all intracellular contaminants and focus on the secreted proteins of the invasion front. With this filter we identified 152 proteins (depicted as “TRUE” in the prediction column of Supplementary Table 7). Of these, 98 were predicted as non-membraneous and 54 as membraneous, spanning a number of transmembrane helices from 1 to 10. Of the non-membraneous proteins, 75 were predicted as secreted with the classical secretory pathway and 23 with the non-classical one (Supplementary Table 7). Since this 152-protein dataset represents a dataset of secreted proteins originating from tumor host cell interactions, we assigned it with the term “Desmoplastic Protein Dataset” (DPD) (Supplementary Table 8).

### Smooth muscle enrichment analysis in DPD

**Figure 4:**
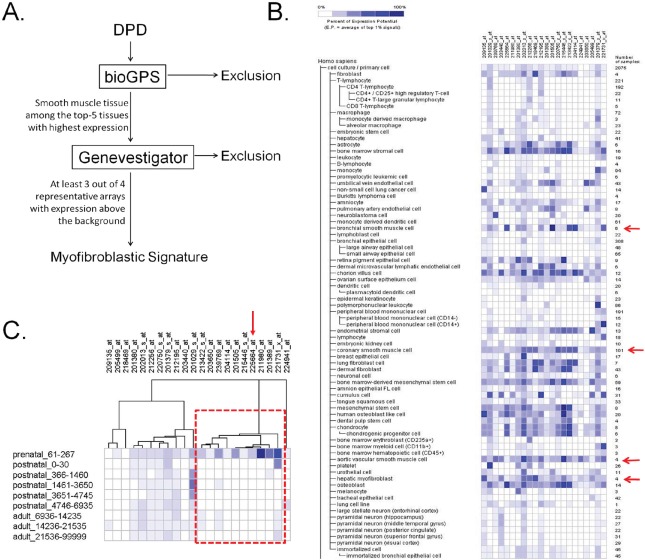
Myofibroblastic signature (A) Algorithm of smooth muscle enrichment analysis. For details see text. The algorithm retrieves smooth muscle-expressed and/or -specific proteins, as potential myofibroblastic markers. (B) Heat map generated from Genevestigator, showing the expression of the 22 protein subset of the proposed myofibroblastic signature, in various normal cell arrays. Red arrows depict the representative smooth muscle-like cells. Columns represent the 22-gene cluster of the myofibroblastic signature; rows represent normal tissues/cell types in which th gene expression of each probe was investigated. (C) Hierarchical clustering of the myofibroblastic signature across various age groups in *Homo sapiens* arrays revealed a cluster of 10 proteins, which are highly-expressed in prenatal (fetal) arrays, when compared to the adult ones. Columns represent the probes of the 22-gene cluster of the myofibroblastic signature; rows depict the age group in days. The red arrow points to the Collagen type XII (COL12A1) probe.

A consistent feature of peritumoral fibroblasts in desmoplastic lesions is a myofibroblastic phenotype, which is based on the synthesis of intracellular smooth-muscle like proteins, in particular α-SMA [39, 40]. Multiple other markers of myofibroblastic differentiation have been described, most of which are components of the ECM [9]. It is now well-accepted that CAFs deploy their smooth muscle gene and protein expression machinery, which contributes to their enhanced capability of mechano-transduction and migration within the tumorigenic stroma [9]. Thus, we assumed that the DPD could be possibly enriched in secreted markers of myofibroblastic differentiation. We created a rationalized gene-expression meta-analysis strategy, termed as “smooth muscle enrichment analysis”, to investigate such proteins. The meta-analysis criteria (Figure 4A) included proteins of the DPD that were highly expressed in smooth muscle (e.g. bronchial smooth muscle) or smooth muscle-like (e.g. hepatic myofibroblast) arrays, independent to the organ of origin (i.e. not necessarily in colonic tissues). Our results designated 22 proteins as smooth muscle-expressed or -specific (Figure 4B, Table 1), which is a novel, myofibroblastic signature. In addition, there was a tendency for these proteins to be highly expressed in mesenchymal-like arrays, rather than epithelial-like, when assessed in the gene expression meta-analysis heat map (Figure 4B). For instance, bone marrow stromal cell, chorion villus cell, endometrial stromal cell, bone marrow-derived mesenchymal cell and mesenchymal stem cell expression was significantly high for most of these proteins. In contrast, transcript expression of these candidates was particularly low in arrays corresponding to immune cells (monocytes, B-lymphocytes, T-lymphocytes, dendritic cells), bone marrow haematopoietic lineages, as well as epithelial-like arrays (tracheal, bronchial, lung, epidermal keratinocyte, tongue squamous cell). Finally, using Gene Enrichment Profiler (see Methods), we validated that all proteins of the myofibroblastic signature had very high enrichment scores in smooth muscle arrays (Supplementrary Figure 4).

**Table 1:** 

Gene Name	Protein Name	Affymetrix Probe	Myofibroblastic Signature	Fetal-like Signature
ASPH	Aspartyl/asparaginyl beta-hydroxylase	209135_at	yes	no
CD99	Isoform I of CD99 antigen	201029_s_at	yes	no
CDH11	Cadherin-11	239769_at	yes	yes
CDH2	Cadherin-2	203440_at	yes	no
COL12A1	Isoform 4 of Collagen alpha-1(XII) chain	225664_at	yes	yes
COL4A1	Isoform 1 of Collagen alpha-1(IV) chain	211980_at	yes	yes
CRTAP	Cartilage-associated protein	201380_at	yes	no
EXT2	Isoform 1 of Exostosin-2	202013_s_at	yes	no
GALNT10	Isoform 1 of Polypeptide N-acetylgalactosaminyltransferase 10	212256_at	yes	no
GREM1	Isoform 1 of Gremlin-1	218468_at	yes	no
IL6ST	Isoform 1 of Interleukin-6 receptor subunit beta	212195_at	yes	no
ITGA5	Integrin alpha-5	201389_at	yes	yes
LAMB1	LAMB1 200 kDa protein	201505_at	yes	yes
LEPRE1	Isoform 3 of Prolyl 3-hydroxylase 1	220750_s_at	yes	no
LOX	Protein-lysine 6-oxidase	215446_s_at	yes	yes
MXRA8	Isoform 2 of Matrix-remodeling-associated protein 8	213422_s_at	yes	yes
NID2	Nidogen-2	204114_at	yes	yes
PAPPA	Pappalysin-1	224941_at	yes	no
PROCR	Endothelial protein C receptor precursor	203650_at	yes	yes
SRPX2	Sushi repeat-containing protein SRPX2	205499_at	yes	no
TPD52L2	Isoform 2 of Tumor protein D54	201379_s_at	yes	no
VCAN	Isoform V0 of Versican core protein	221731_x_at	yes	yes

We further compared the relative expression of these 22 proteins in various prenatal and postnatal arrays, allowing us to classify them into distinct clusters with specific gene expression patterns in fetal tissues. This analysis revealed a small cluster of 10 proteins (Figure 4C red box, Table 1), which was highly expressed in fetal tissue arrays compared to adult ones. This could be an interesting observation, since it is now speculated that CAFs obtain two potential phenotypes at the tumor-host cell interface area, the myofibroblastic phenotype and the fetal-like phenotype [9]. Initially it was shown that the latter subpopulation was exclusively present during developmental processes, but later, it became evident that fetal-like fibroblasts participate in wound healing processes in adult tissues, as well [41]. Thus, fetoprotein cluster analysis could specify secreted, fetal-like markers in DPD, which could characterize the tumor invasion front.

### Collagen type XII is as a novel marker of myofibroblastic differentiation in colorectal cancer desmoplastic lesions

Of all the candidates of the myofibroblastic signature, we turned our focus to collagen type XII, for the following reasons: (A) Various ECM components deposited in desmoplastic lesions, including collagens, have been widely demonstrated to be markers of myofibroblastic differentiation [9]. (B) Collagen type XII has been previously linked to other fibrotic diseases [42, 43], but never specifically to cancer-associated desmoplasia. (C) Data obtained from our proteomic analysis suggests that it is extremely unlikely that collagen type XII could be a false positive protein (it was identified with a very large number of peptides; 68 in SW480Co and 92 in SW620Co). (D) Collagen type XII has been previously demonstrated by proteomics to be expressed by smooth muscle cells, mainly perivascularly. In this global proteomic approach [44], a mass spectrometry-based platform for identification of vascular extracellular proteins in human aorta samples, revealed a novel ECM component signature, including various proteoglycans and collagens (such as collagen type XII). (E) Finally, collagen type XII also belonged to the fetal-like-generated subsignature (probe 225664_at, Figure 4C, Table 1), thus it could be associated with the phenotype of fetal-like CAFs, giving additional strength to its prioritization. For these reasons, we investigated collagen type XII expression with immunohistochemistry in colorectal cancer desmoplastic lesions.

**Figure 5:**
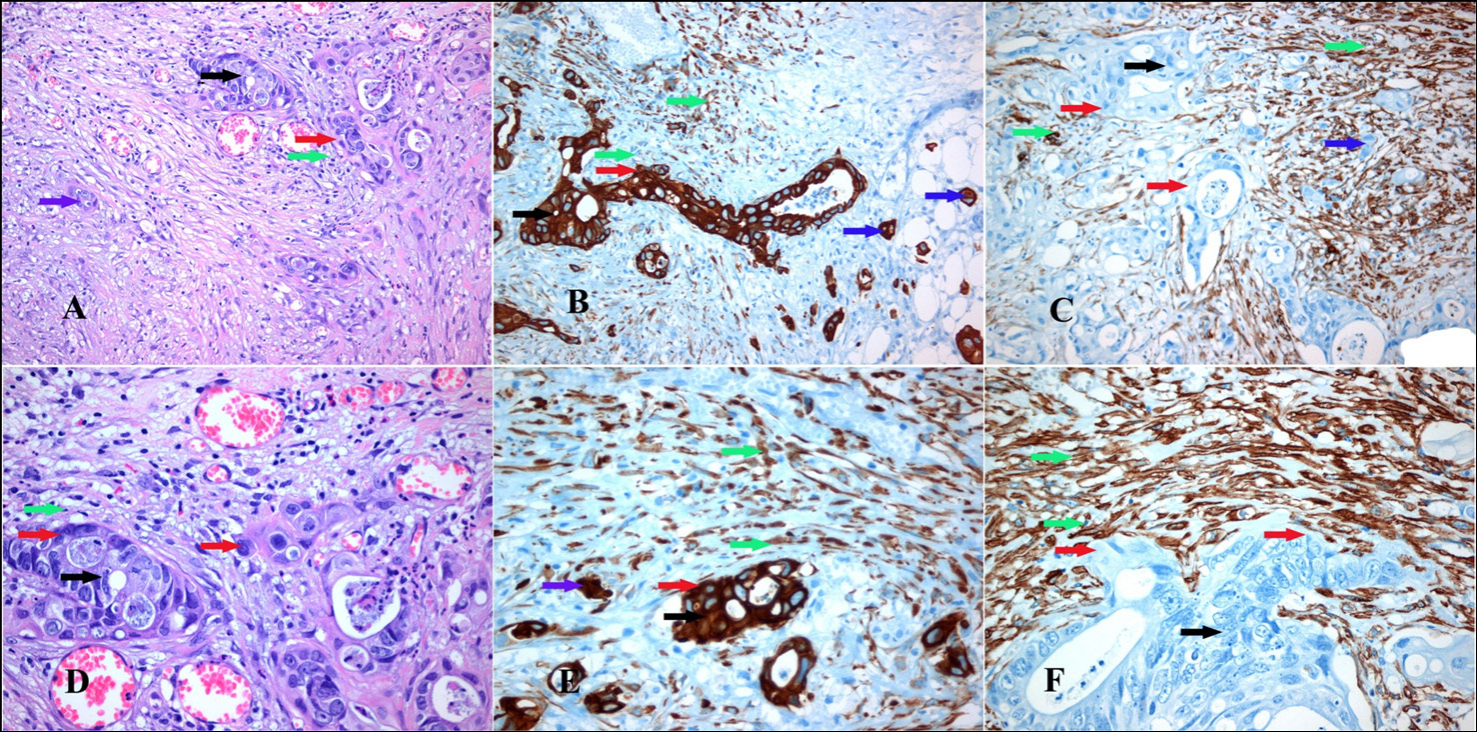
Immunohistochemical markers defining the cell populations of the tumor invasion front Tumor invasion front area (red arrows) of colorectal carcinoma (black arrows) with myofibroblastic stromal reaction (green arrows) containing tumor budding cells (blue arrows). (A & D) H-E stain. (B & E) Pan-keratin immunoexpressed strongly in CRC cells and slightly in myofibroblasts. (C & F) alpha-SMA immunoexpressed in myofibroblastic stromal reaction. *Magnifications: A, B & C x 200, D, E & F x400*.

**Figure 6:**
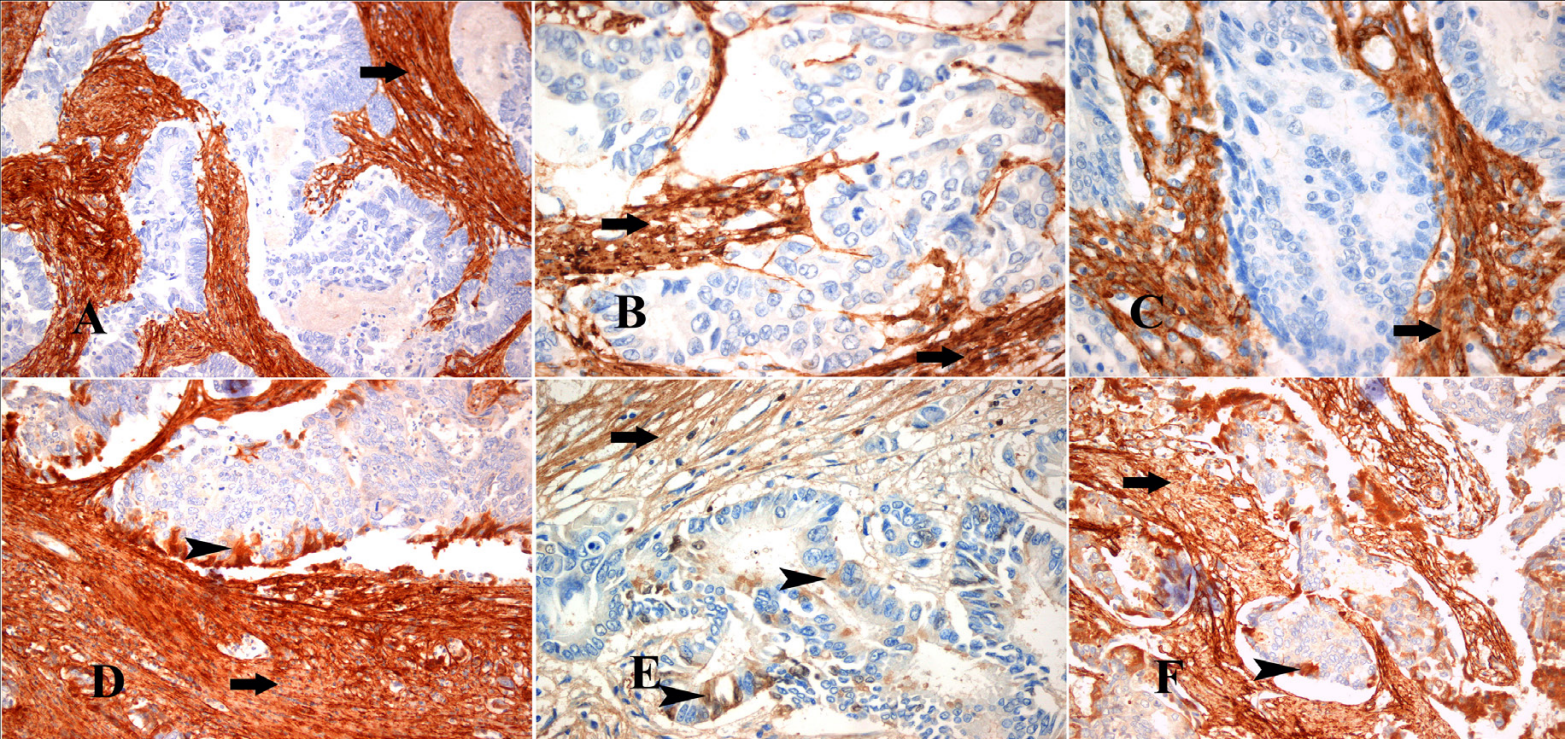
Immunohistochemical profile of the myofibroblastic stromal reaction in areas of invasion of CRC (A-C) Collagen III immunohistochemical expression in the stromal collagen and myofibroblasts (arrows) around the tumor invasion front in cases of CRC (D-F) Collagen XII immunohistochemical expression in the stromal collagen and myofibroblasts (arrows) around the tumor invasion front in the same cases of CRC, respectively, and in tumor cells of the tumor invasion front (arrowheads). *Magnifications: A, D, F x200 and B, C, E x400*.

First, we defined the cancerous and stromal subpopulations at invasive fronts of desmoplastic lesions in a small cohort of CRC patients (Figure 5). In such areas, the stromal reaction consisted of collagen, CAFs and inflammatory cells (Figures 5A&D). Cancer cells invading the stroma were characterized by strong keratin expression (Figures 5B&E). A strong cytoplasmic α-SMA immunohistochemical expression was observed in the myofibroblasts around CRC invasive areas (Figures 5C&F). Subsequently, we investigated collagen expression in these samples. Both collagen type III and XII were strongly co-immunoexpressed in the collagenous matrix around tumor cell cohorts, as well as in CAFs (Figures 6A-E). Thus, our data strongly support the notion that collagen type XII could be an indicator of myofibroblastic induction in CRC desmoplastic lesions.

### Colon cancer cells, in addition to CAFs, could potentially contribute to the secretion of collagen type XII at the desmoplastic invasion front

**Figure 7:**
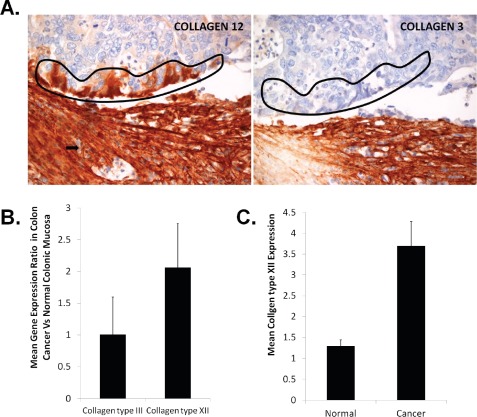
Investigation of Collagen type XII expression in colon cancer cells (A) Cells lining the invasion front (encircled) and tumor budding cells (arrows) were found positive for collagen type XII and negative for collagen type III. (B) Mean ratios of Collagens type III and XII, calculated from gene expression meta-analysis between colorectal cancer adenocarcinomas and normal colonic mucosa in a total of 6 experiments in the Genevestigator database. (C) Mean COL12A1 expression in cancerous lesions of 24 CRC patients and adjacent to the tumor normal colonic mucosa. *All bars are mean values with standard deviations calculated from all experiments or replicates*.

One finding from our analysis was the expression of collagen type XII by some cancer cells at the interaction line (Figures 6D-F, Figure 7A), and additionally by tumor buds (Figure 7A). Tumor cells positioned in the core of the cohort were negative for collagen type XII (Figures 6D-F). This was not the case for collagen type III, since this type of collagen was characteristic of the stromal compartment, exclusively (Figures 6A-C, Figure 7A). Based on these observations, it was tempting to speculate that collagen type XII, in addition to being a potential myofibroblastic marker, it could also serve as marker of the tumor invasion front cancer cells.

We further investigated whether there was any evidence of collagen type XII expression by colon cancer cells. First, by using gene expression meta-analysis of cancer microarrays (Genevestigator), we identified six experiments from two studies [45, 46], five of which depicted comparisons of gene expression patterns between colon cancer and normal colon mucosa tissues (Supplementary Figure 5). We calculated mean gene expression levels for the probes corresponding to both collagens and found that collagen type XII is ~2-fold increased, while collagen type III expression was not significantly altered in colon cancer cells compared to normal colonic mucosa (Figure 7B, Supplementary Figure 5). We validated the collagen type XII meta-analysis results in a cDNA array, consisting of 24 CRC patients, matched with normal colonic mucosal tissue adjacent to the tumor (Supplementary Figure 6). This analysis revealed a ~3.8-fold overexpression (p<0.05) of collagen type XII mRNA levels in cancerous tissue compared to normal colon mucosa (Figure 7C). However, these data should be interpreted with caution since stromal contamination occurs to some extent upon sampling epithelial cancers invading the stroma. Taken together, we provided evidence that collagen type XII might be produced by cancer cells at the desmoplastic invasion front, in addition to CAFs.

## DISCUSSION

We applied a mass spectrometry-based proteomic strategy to investigate tumor-host cell interactions, based on proteomic analysis of mono- and coculture CM. We particularly sought to identify novel regulatory mechanisms of carcinogenesis in desmoplastic invasion fronts of colorectal cancer. Such high-throughput strategies may provide new insights into the regulation of cancer-related phenotypes that are microenvironmentally determined [47]. It should be noted that our *in vitro* model system involved only two colon cancer cell lines (SW480 and SW620), which were derived from a different stage of the same patient [29]. Thus, the proteins of the DPD (Supplementary Table 8) may have been influenced by the specific genetic backgrounds of these two colon cancer cell lines. For instance, it is known that SW480 is deficient in SMAD4, although it is responsive to TGF-β signaling [48, 49]. Therefore, the non-canonical TGF-β signaling in the SW480 mono- and cocultures could be partially responsible for the identified subproteomes. Although, the inclusion of many cell lines could probably resolve such issues, this also entails additional cost. Since our approach depicts a proof-of-concept study, we conclude that the DPD should be evaluated with caution, as it represents only a proteomic snapshot of the dynamic interactions of the tumor invasion front.

Of considerable importance in this study, is the identification of the *desmoplastic protein dataset* (Supplementary Table 8). This is the first characterization of a proteomic dataset that corresponds to candidate secreted proteins of the “cancer invasion front”. One limitation is our inability to distinguish whether a protein of the DPD originated from the cancer cells or the CAFs, in the coculture conditions. Since none of these proteins were identified via mass spectrometry in any of the monocultures, their presence in the coculture CM could be attributed to the tumor host cell interactions, such as paracrine signaling through secreted or cell-adhesion factors. However, it is possible that many low-abundance proteins (e.g. cytokines) might not have been identified in a monoculture dataset, due to method sensitivity. Thus, the DPD is prone to false positives. For this reason, proteins that were considered as candidate regulators of tumor-host cell interactions, were subjected to both stringent criteria of identification (at least 2 peptides) and validation analyses, including IHC.

An examination of the DPD reveals the paradoxical co-existence of positive and negative regulators of carcinogenesis. It remains obscure whether the desmoplastic microenvironment plays a tumor-promoting or a tumor-suppressive role in colorectal cancer, and the literature is divided [9]. On one hand, the desmoplastic reaction represents an effort of the healthy tissue to entrap and encircle the tumor, by providing a biological limitation to the expansion of cancer cells. However, cancer cells seem to adapt in this continuously modified desmoplastic landscape and develop alternative pathways to shift desmoplastic proteins towards their own favour [50, 51]. In general, the host responses to neoplastic lesions seem to represent a paradox. This is also evident in cancer-associated inflammation, whose primary role is to suppress cancer. However, it is now well-established that chronic inflammation in cancerous microenvironments is shifted towards promoting cancer progression [19, 31, 52].

By utilizing a series of bioinformatic tools, we generated and partially validated a proteomic myofibroblastic signature (Figure 4B) with potential functional importance in the regulation of colorectal cancer metastasis. The smooth muscle enrichment analysis, proposed here for the first time, unraveled markers of myofibroblastic differentiation. By focusing on several smooth muscle-like arrays in various gene-expression profile databases, we selected proteins characterizing the myofibroblast proteome, since it is well-demonstrated that CAFs deploy a full smooth muscle-like expression program. Notably, this strategy allowed us to re-identify previously-reported markers of myofibroblastic differentiation, such as the proteoglycan versican (VCAN) isoforms V0 and V1 [53, 54], which validated our selection criteria. Interestingly, the myofibroblastic signature consisted of many desmoplastic proteins, e.g. constitutents of the ECM, as well as ECM-remodelling enzymes. Indeed, many markers of myofibroblastic differentiation are proteins that are secreted by CAFs and deposited peritumorally (at the cancer invasion front) to form a desmoplastic film, as a reaction of the host tissue towards the invading cancer cells [9].

Collagen type XII was found in the myofibroblastic signature, but never reported before to be secreted by CAFs. Collagen type XII belongs to the fibril-associated collagens with interrupted triple helices (FACITs), which are, in fact, fibrillar collagen organizers. Collagen type XII (as well as its relevant collagen type XIV) has one domain that anchors the molecule to the surface of another fibril (e.g. collagens type I and III), and three “finger-like” domains [55]. By residing in collagen I fibrils, collagen type XII participates in fibril structure interaction and organization, by further stabilizing them [56]. It has recently been reported that desmoplastic lesions are usually characterized by non-organized collageneous fibril deposition around tumor invasive cohorts, which significantly differs from normal collagen [57-59]. This loss of smooth collagen reorientation, and acquisition of a more disorganized one, called ‘cancer-associated collagen’, is very characteristic during cancer development [60, 61]. Therefore, it is tempting to speculate from our data that CAFs are recruited by cancer cells in an attempt to re-organize the cancer-associated collagen in desmoplastic lesions through various mechanisms, including the secretion of collagen type XII. However, additional experiments are needed to unravel the regulation of its secretion, as well as its exact role in tumor progression.

**Figure 8:**
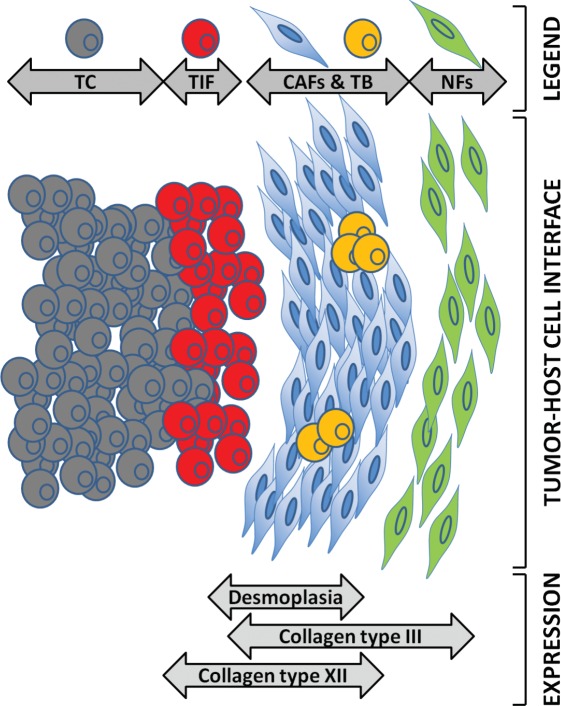
Proposed model for the expression of collagen types III and XII at the CRC desmoplastic tumor microenvironment The cartoon in the middle shows the tumor host cell interface; rounded cells represent cancer cells; spindle-shaped cells represent stromal cells. Colors show different cell subpopulations as depicted in legend. TC: tumor core cancer cells (grey, rounded); TIF: tumor invasion front cancer cells (red, rounded); TB: tumor budding cancer cells (orange, rounded); CAF: cancer-associated fibroblasts (blue, spindle-shaped); NF: normal fibroblasts (green, spindle-shaped). The gray double-edged arrows demonstrate where the expression of various markers (such as collagens) expands relatively to the tumor host cell interface.

Our *in vitro* experiments revealed that collagen type XII was abundantly secreted in both SW480Co and SW620Co cocultures, but we could not discern which cell type was responsible for this production. Immunohistochemical analysis revealed its expression by cancer cells lining the desmoplastic front and by cancer cells of the tumor budding subpopulation, in addition to CAFs. It is interesting that Collagen XII, in contrast to Collagen type III, was shown not to be just another stromal marker in our analysis. It is widely known that cancer cells progressively lose their differentiation upon reaching the tumor invasion front area and, especially, tumor budding [4, 6, 62]. These observations support the notion that collagen type XII might also be secreted by cells undergoing a dedifferentiation program, such as EMT, which is another reported mechanism of myofibroblastic induction [7]. Taken together, all these observations could contribute to a better understanding of the tumor-host cell interface in the CRC desmoplastic invasion front. Based on all these findings, we propose a schematic model of collagen expression at the desmoplastic invasion front in CRC metastasis (Figure 8).

## METHODS

### Antibodies

The following antibodies were purchased: mouse monoclonal anti-CK (cytokeratin cocktail) from Cell Marque (clone A1E&AE3), mouse monoclonal anti-alpha-SMA from DAKO (clone 1A4), mouse monoclonal anti-collagen-III from Biogenex (clone HWD1.1; concentrated in PBS), mouse monoclonal anti-collagen-XII from Sigma-Aldrich (clone 1303). Antibodies against the kallikreins 6 (KLK6), 7 (KLK7) and 10 (KLK10) were rabbit polyclonal, generated in our laboratory [63].

### Cell culture

#### Cell lines

The human SW480, SW620, HCT116 and HT29 colon cancer and CCD18Co (18Co) normal colonic fibroblast cell lines were purchased from the American Type Culture Collection (ATCC, Rockville, MD) and were maintained in their favorable media, according to the manufacturer's instructions. All experiments were performed within the first 10 passages from the initiation of all cultures.

#### Stimulation experiments

For the preparation of conditioned media (CM) for stimulation experiments, 18Co cells were cultured in T-175 flasks to ~75-80% confluency and then switched to serum-free medium (chemically defined Chinese hamster ovary – CDCHO) for 2 additional days. Then, CM were harvested and centrifuged at 1,500 rpm for 5 min to remove cellular debris. CM were then concentrated 4 times, using a 5 kDa molecular weight cutoff (Millipore Pellicon XL) and rediluted in fresh CDCHO to the initial volume. CM were filter-sterilized using a 0.22 μm membrane cut-off (Millipore) and kept at -20 °C until used for stimulation experiments. All frozen CM were used within 15 days from their preparation.

#### Development of cocultures

The cell-contact desmoplastic cocultures SW480/18Co, SW620/18Co, HT29/18Co and HCT116/18Co were termed as SW480Co, SW620Co, HT29Co and HCT116Co, respectively. 18Co fibroblasts were initially seeded at T-175 flasks and allowed to reach at least 50% confluency in minimum essential medium (MEM), supplemented with 10% fetal bovine serum (FBS). Then, flasks were rinsed with PBS to remove dead cells. Colon cancer cells (2X106) were resuspended in Dulbeco's modified Eagle medium (DMEM) plus 10% FBS and placed on top of the pre-seeded 18Co cells. The cocultures were left to grow on DMEM 10% FBS for at least 2-3 additional days prior to further experiments. 18Co cells showed excellent viability when cultured in either MEM or DMEM with 10% FBS, using a trypan blue assay (data not shown).

### Proteomic analysis of CM

#### Sample preparation

All mono- and cocultures were washed with PBS and switched to CDCHO medium for 2 days. All CM were collected and normalized to a total of ~1.1 μg of total protein (Coomassie colorimetric assay; Pierce biotechnology); samples were dialyzed, using a 3.5 kDa molecular cut-off membrane (Spectrum Laboratories, Inc., CA, USA), in 4 L of 1 mM ammonium bicarbonate solution (Fisher Scientific) (4 buffer changes). Dialyzed CM were frozen at −80 °C and subjected to lyophylization until total dryness; samples were then denaturated by 8 M urea (Fisher), reduced with dichloro-diphenyl-trichloroethane (DDT) (Sigma-Aldrich) to a final concentration of 13 mM at 50 °C for 30 min, alkylated with 500 mM iodoacetamide (Sigma-Aldrich) in the dark, at room temperature for 1 h, and finally desalted using a NAP5 column (GE Healthcare), using the manufacturer's instructions. The 1 mL eluted samples were lyophilized and trypsin-digested (Promega) at a molar ratio of 1:50 (trypsin:protein concentration) for 8 h. The peptides were again lyophilized to dryness and resuspended in 120 μL of 0.26 M formic acid in 10% acetonitrile (mobile phase A).

#### Strong cation exchange liquid chromatography (SCX-LC)

The samples were fractionated using an Agilent high-performance liquid chromatography (HPLC) system connected to a PolySULFOETHYL ATM column with 200-Å pore size and a diameter of 5 μm (The Nest Group Inc.). A 1-h linear gradient was used with 1 mol/L ammonium formate and 0.26 mol/L formic acid in 10% acetonitrile (mobile phase B) at a flow rate of 200 μL/min. Fractions were collected every 5 min for the first 20 min of the run and every 2 min for the following 40 min, to a total number of 24 fractions/replicate. Of these, 16 fractions, corresponding to the highest concentration of eluted peptides, were kept for mass spectrometry. A peptide cation exchange standard (Bio-Rad), consisting of four peptides, was run at the beginning of each replicate to assess column performance.

#### Reverse phase liquid chromatography (RP-LC)

HPLC fractions were C18-extracted using a ZipTipC18 pipette tip (Millipore) and eluted in 5 μL of 90% acetonitrile, 0.1% formic acid, 10% water and 0.02% trifluoroacetic acid (Buffer B). 80 μL of 95% water, 0.1% formic acid, 5% acetonitrile and 0.02% trifluoroacetic acid (Buffer A) were added to this mixture, and 40 μL were injected via an autosampler on an Agilent HPLC. The peptides were first separated onto a 2-cm C18 guard column (inner diameter 200μm), then eluted onto a resolving 5-cm analytical C18 column (inner diameter 75 μm) with an 8-μm tip (New Objective).

#### Tandem mass spectrometry (MS/MS)

This HPLC system was coupled online to an LTQ-Orbitrap XL (Thermo Fisher Scientific) mass spectrometer, using nano-electrospray ionization (ESI) source (Proxeon Biosystems), in data-dependent mode. Each fraction was run with a 55-min gradient and eluted peptides were subjected to one full MS scan (450-1450 *m/z*) in the Orbitrap at 60,000 resolution, followed by six data-dependent MS/MS scans in the linear ion trap (LTQ Orbitrap). Unassigned charge states and charges 1+ and 4+ were all ignored, as depicted through the charge-state screening and preview mode.

#### Protein identification

Data files were created by use of Mascot Daemon (version 2.2.0) and extract_msn. The resulting mass spectra from each fraction were analyzed using Mascot (Matrix Science; version 2.2) and X!Tandem (Global Proteome Machine Manager; version 2005.06.01) search engines, using the International Protein Index human database (version 3.62, 167,894 protein sequences), which includes both forward and reverse sequences. The resulting Mascot and X!Tandem files were loaded into Scaffold (Proteome Software; version 2.6) to cross validate the data files from both engines. Detection of a minimum of two unique peptides was required to accept positive protein identification. A normalized method for spectral counting analysis was used and spectrum reports were exported from Scaffold and uploaded into an *in-house* developed database (http://www.acdc-proteome.info/) for further analysis. This database provides the tools to compare protein datasets and generate Venn diagrams from proteomic experiments.

### Bioinformatic analysis

#### Selection of secretome proteins

Individual proteins were subjected to prediction of protein secretory pathway with the presence of signal peptide (classical secretory mechanism) (SignalP 3.0) (http://www.cbs.dtu.dk/services/SignalP/) [64, 65] and without the presence of signal peptide (non-classical secretory mechanism) (SecretomeP 2.0) (http://www.cbs.dtu.dk/services/SecretomeP) [66] and for presence of amino acid sequences that correspond to transmembrane helices (TMHMM 2.0) (http://www.cbs.dtu.dk/services/TMHMM) [67]. The sequential use of the above-mentioned tools on a given proteomic dataset, is termed “secretome algorithm”.

#### Smooth muscle enrichment analysis

Two publicly-available gene expression meta-analysis databases were utilized to examine the tissue and cell type expression patterns of individual proteins of interest. These databases (Genevestigator and bioGPS) allow the user to study gene expression patterns from multiple microarray experiments. Since we wished to define smooth muscle-like and/or -specific proteins as potential myofibroblastic markers of CAFs, we termed this gene expression meta-analysis, as “smooth muscle enrichment analysis”. For Genevestigator (https://www.genevestigator.com/) [68], only probes (1-3 per protein) with high probability to correspond to the actual gene of interest were searched for their tissue expression profiles. For some proteins, no such probes were found, and these were excluded from the meta-analysis. A heatmap showing expression of these genes in various human, primary, normal and cancer cell lines was constructed based on microarray data from both gene expression omnibus (GEO) (5146 microarray experiments) and ArrayExpress (54 microarray experiments) array sources. Genevestigator was set to automatically accept high quality control arrays in the meta-analysis. We observed that the normal cell line arrays, in Genevestigator, included four smooth muscle-like cell types: bronchial smooth muscle cell, coronary smooth muscle cell, aortic vascular smooth muscle cell and hepatic myofibroblast. Although none of these arrays actually corresponds to colonic tissues and no colonic myofibroblast arrays currently exist in any database, we accepted the notion that these 4 array types could be representative of any smooth muscle-like tissue, if the gene expression pattern was reproducible. Thus, we accepted as “smooth muscle-like proteins”, only those that had high expression levels in at least three out of these four representative arrays, in the Genevestigator database. To achieve higher confidence of smooth muscle-like and/or -specific proteins, we integrated expressional data from Genevestigator with the bioGPS database. In the latter database, we accepted proteins that did not have a wide expression pattern; thus we accepted only those with significant expression in less than 5 of the bioGPS reported tissues, compared to the background expression levels. In addition, we accepted only those proteins whose expression in the “smooth muscle tissue” arrays was amongst the 3 highest expressions in all bioGPS arrays.

#### Tissue specificity

We investigated the tissue specificity of individual gene probes of interest with Gene Enrichment Profiler (http://xavierlab2.mgh.harvard.edu/EnrichmentProfiler/), containing 126 human primary tissues represented by 557 microarray experiments. Enrichment graphs were exported directly from the application.

### Immunocytochemistry

18Co cells were plated on poly-L-lysine-coated coverslips (POLY-PREP SLIDES, Sigma) at approximately 30,000 cells/coverslip, in MEM, 10% FBS, and were left for 24 h in Petri dishes to allow for adherence and flattening. Once the fibroblasts adhered, the coverslips were washed with phosphate buffered saline (PBS). SW480 and SW620 cells were plated on top of the fibroblasts at approximately 20,000 cells/coverslip, in DMEM, 10% FBS and were left for 32 h to allow for adherence and proliferation. Then, the slides were washed with PBS and left for an additional 72 h, in CDCHO, to allow growth and paracrine signaling. Replicates of monocultured cells, served as controls. After the termination of cocultures, all slides were washed with PBS, fixed in 4% paraformaldehyde for 1 h and soaked in ethanol overnight. The cells were permeabilized, using 2 mL of 0.05% Triton-X-100 detergent for 5 min. Cells were washed with PBS and covered with 0.5 mL peroxidase blocking reagent for 5 min. Cells were again washed with PBS and subsequently incubated for 1.5 h at room temperature with primary antibody, directed against α-SMA, diluted 1:50 in PBS. Cells were then washed with PBS and incubated with 0.5 mL goat serum blocking agent for 15 min. cells were washed again with PBS and incubated with peroxidase-conjugated secondary antibody, diluted 1:200, in PBS. Cells were washed again with PBS and incubated with 0.2 mL DAB chromogen buffer and counterstained with hematoxylin, following routine staining procedures. Replicates of no-antibody controls were also included. Fluorescence was visualized using light microscopy (Olympus), attached to a Q-Color3 camera (Olympus) and Q-Capture Pro image analysis software.

### Immunohistochemistry

Enrolled were paraffin tissue sections from 15 archived cases of moderately or poorly differentiated human CRC, showing an intense desmoplastic reaction in invasive areas. The immunohistochemical staining was performed using the Bond automated immunohistochemistry system (Bondmax, Leica Microsystems, UK)-pretreatment with epitope retrieval (pH 8). The following mouse monoclonal antibodies were used; against Cytokeratin cocktail (Cell Marque, clone A1E&AE3, 1:70), alpha-SMA (DAKO, clone 1A4, 1:100), Collagen III (Biogenex, clone HWD1, 1:100) and Collagen XII (Sigma-Aldrich, clone 1303, 1:30).

### *In-vitro* functional assays

#### Cell proliferation (crystal violet) assay

Cancer cells (SW480, SW620) were plated in 24-well plates at low confluency (~25%) and left for 24 h in DMEM 10% FBS to allow for adherence. Then, all wells were washed with PBS and specific experimental conditions were applied in each well. After the termination of the experiment, all CM were removed, cells were washed with PBS and subsequently fixed with 10% formalin for 20 minutes. After fixation, crystal violet solution (0.05%) was applied in the wells (~1 mL) for 30 minutes. For quantitative assessment, 1 mL of 100% methanol was added in each well to solubilize the absorbed crystal violet dye, plates were gently shaken for 3 minutes and absorbance was measured at 540 nm. Data were graphed as relative mean absorbance from a total of six replicates per condition.

#### Cell migration (scratch) assay

Cancer cells were seeded at six-well plates. After forming confluent monolayers, a pipette tip was used to create a single straight scratch on the well and they were rinsed 3 times with 4-5 mL of PBS to remove detached and dead cells. After washing, the experimental conditions and treatments were applied in the wells, in at least 3 biological replicates. The wells were left for 36 hours then all media were removed, cells were washed once with PBS and fixed with 10% formalin for 20 minutes. Cells were washed twice with PBS and examined under light microscopy.

#### Cell adhesion assay

Our preliminary observations show that SW480/SW620 cells are not able to strongly adhere in cell culture plates without FBS; however, if these cells are already attached, a subsequent serum deprivation does not cause detachment, even if it persists for 6-7 days (data not shown). Based on this, we developed a simple cell adhesion assay setup. SW480/SW620 cells were resuspended in serum-free medium, and reseeded in 24-well plates. Then, various experimental conditions were applied in the wells; in some cases, plates have already been pre-seeded with 18Co fibroblasts (20% confluency) and in other cases only 18Co medium was used as a supplement in the wells. Untreated wells served as controls. Adhesion of cancer cells on the plate surface or pre-seeded fibroblasts was estimated after 48 hours, using light microscopy.

#### Resistance to chemotherapy assay

SW480/SW620 cells were seeded at 5,000/well in 96-well plates and left for 24 hours to adhere and proliferate in DMEM, supplemented with 10% FBS. Then, wells were switched to CDCHO medium and were treated with 5-fluororacil (5-FU) antineoplastic agent in a dose-response manner. For each concentration of the drug, cells were either treated additionally with 18Co or placebo CM. Cells were left for 48 hours to investigate whether the 18Co CM would cause a pharmacological rescue to the drug treatment. The evaluation of cell viability at the termination of the assay was done with the Alamar Blue assay. Specifically, all wells were washed with PBS and Alamar Blue (100 μL of 10% v/v in PBS solution) was added in each well. The reagent was left for 3-4 hours and then fluorescence was measured with fluorescence spectrophotometer using 560 EX nm/590 EM nm filter setting. Alamar blue is a stable, soluble, non-toxic agent that monitors the innate metabolic activity of the cells. The assay is based on an oxidation-reduction indicator, directly proportional to metabolic activity, thus “translating” the fluorescence signal into viability signal.

### ELISA

The concentration of KLK6, KLK7 and KLK10 in mono- and coculture supernatants, was determined by *in-house* developed sandwich-type immunoassays, as previously described [63]. Concentration was determined by interpolation from a standard curve, using recombinant proteins, as standards. The concentrations of MMP2 and urokinase-type plasminogen activator (uPA) were measured with commercially available immunoassays (Invitrogen).

### Quantitative PCR

For clinical validation, the TissueScan Colon Cancer cDNA Array III was used (Origene). Quantitative PCR was conducted on these samples using 1X SYBR green reagent (Applied Biosystems) and transcript levels of beta-actin and COL12A1 were measured on a 7500 ABI System. The following forward (F) and reverse (R) primer sequences were used:

B-actin F 5'- CACCATTGGCAATGAGCGGTTC-3'

B-actin R 5'- AGGTCTTTGCGGATGTCCACGT-3'

COL12A1F5'- GTGCCTGTAGTCAGCCTGAA-3'

COL12A1R5'- GTCTTGTTGGCTCTGTGTCCT-3'

### Statistical analysis

All graphs are presented with mean values and standard deviations calculated from biological replicates. Statistical significance was examined by student's t-test and p<0.05 was considered significant. The statistical software EpiInfo version 7.0.8.0 was used.

## SUPPLEMENTARY TABLES AND FIGURES



















## ABBREVIATIONS

CAF: cancer-associated fibroblast
CDCHO: chemically defined Chinese hamster ovary
CK: cytokeratin
CM: conditioned media
CRC: colorectal cancer
DDT: dichloro-diphenyl-trichloroethane
DMEM: Dulbecco's modified Eagle medium
DPD: desmoplastic protein dataset
ECM: extracellular matrix
EMT: epithelial-to-mesenchymal transition
ESI: electrospray ionization
FACIT: fibril-associated collagen with interrupted triple helices
FBS: fetal bovine serum
5-FU: 5-fluororacil
HGF: hepatocyte growth factor
HPLC: high-performance liquid chromatography
ICC: immunocytochemistry
IGF: insulin growth factor
IHC: immunohistochemistry
IL: interleukin
KLK: kallikrein-related peptidase
MEM: minimum essential medium
MMP: matrix metalloproteinase
MS/MS: tandem mass spectrometry
NF: normal fibroblast
PBS: phosphate buffered saline
RPLC: reverse-phase liquid chromatography
SCX: strong cation exchange
SMA: smooth muscle actin
TGF: transforming growth factor
TNF: tumor necrosis factor
uPA: urokinase-type plasminogen activator
VEGF: vascular endothelial growth factor
