# Supplementary material for: Proteomic Signatures of the Desmoplastic Invasion Front Reveal Collagen Type XII as a Marker of Myofibroblastic Differentiation During Colorectal Cancer Metastasis
Source: Oncotarget. 2012 Mar 8;3(3):267–85. doi: 10.18632/oncotarget.451 (PMC3359884; doi:10.18632/oncotarget.451)
Supplement: Supplementary file 9 [file oncotarget-03-267-s009.pdf]

Proteomic Signatures of the Desmoplastic Invasion Front Reveal Collagen Type XII as a Marker of Myofibroblastic Differentiation During Colorectal Cancer Metastasis.

Karagiannis et al

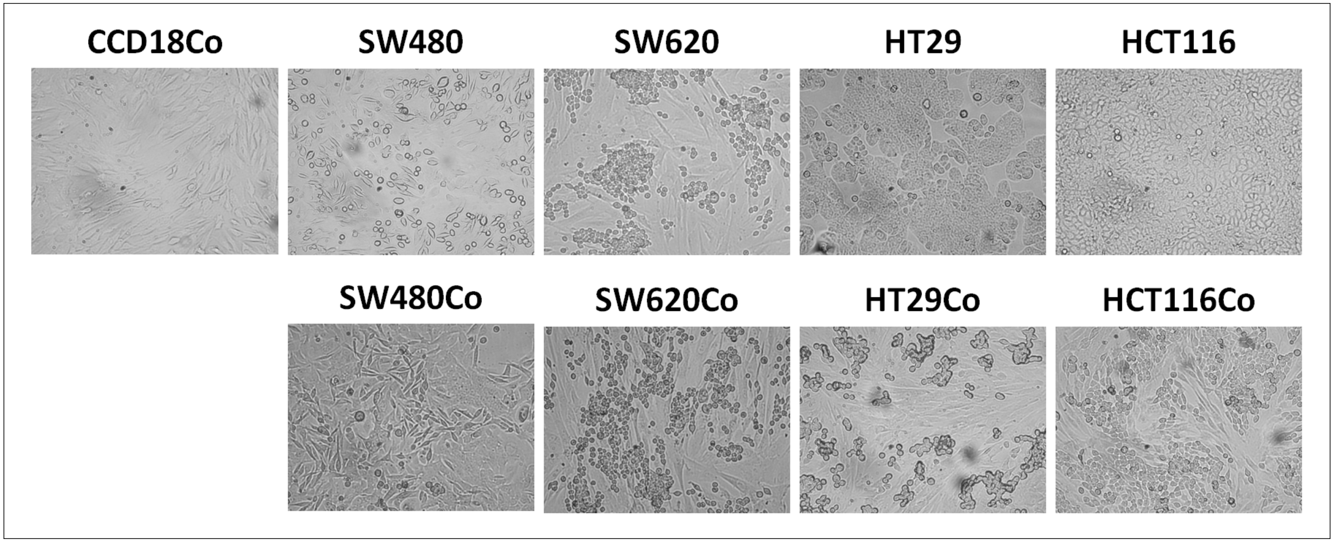

Supplementary Figure 1: Establishment of *in-vitro* co-culture model between colon cancer cell lines (SW480, SW620, HT29, HCT116) and human normal colonic fibroblasts (CCD18Co). The cocultures are named as SW480Co, SW620Co, HT29Co and HCT116Co, respectively. All magnifications x40.

Proteomic Signatures of the Desmoplastic Invasion Front Reveal Collagen Type XII as a Marker of Myofibroblastic Differentiation During Colorectal Cancer Metastasis. Karagiannis et al

Internal control	SW480	SW620	CCD18Co	SW480Co	SW620Co
KLK6	(10,10,12)	(9,8,9)	(1,0,0)*	(8,9,9)	(8,8,7)
	(13,14,16)	(11,11,13)	(1,0,0)	(11,12,14)	(9,10,9)
KLK7	(2,2,3)	(1,0,0)*	(0,0,0)	(0,0,0)	(0,0,0)
	(2,2,3)	(1,0,0)	(0,0,0)	(0,0,0)	(0,0,0)
KLK10	(7,8,6)	(1,1,1)*	(0,0,0)	(6,9,8)	(0,0,0)
	(7,10,9)	(1,1,1)	(0,0,0)	(7,11,10)	(0,0,0)
MMP2	(0,0,0)	(0,0,0)	(16,21,19)	(29,28,33)	(36,32,30)
	(0,0,0)	(0,0,0)	(19,27,26)	(39,47,43)	(49,41,42)

* Internal control not identified because of the minimum of 2 unique peptide hits for protein identification

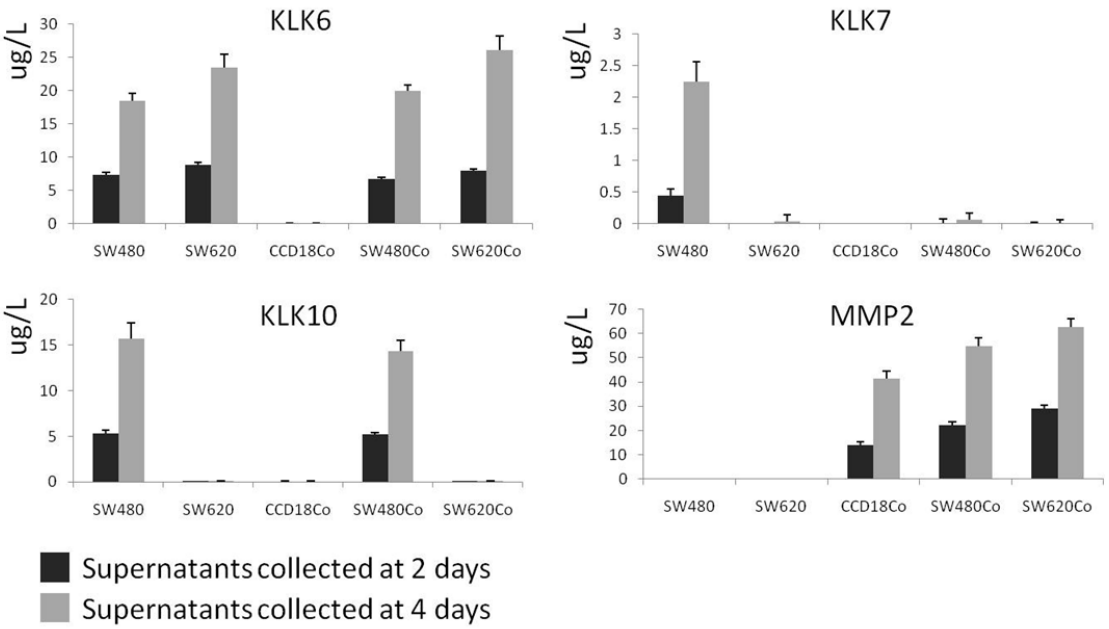

Supplementary Figure 2: Quality control experiment for determination of MS-based protein identification efficiency. A panel of four internal control proteins (KLK6, KLK7, KLK10 and MMP2) was considered and tested for correlation of MS/MS identification (upper panel) with respective immunoassays (lower graphs). Numbers in the upper brackets demonstrate number of unique peptides per identification, in all three replicates, while numbers in lower brackets demonstrate number of unique spectra per identification, in all three replicates. All bars in the graphs represent mean values with standard deviations.

Proteomic Signatures of the Desmoplastic Invasion Front Reveal Collagen Type XII as a Marker of Myofibroblastic Differentiation During Colorectal Cancer Metastasis. Karagiannis et al

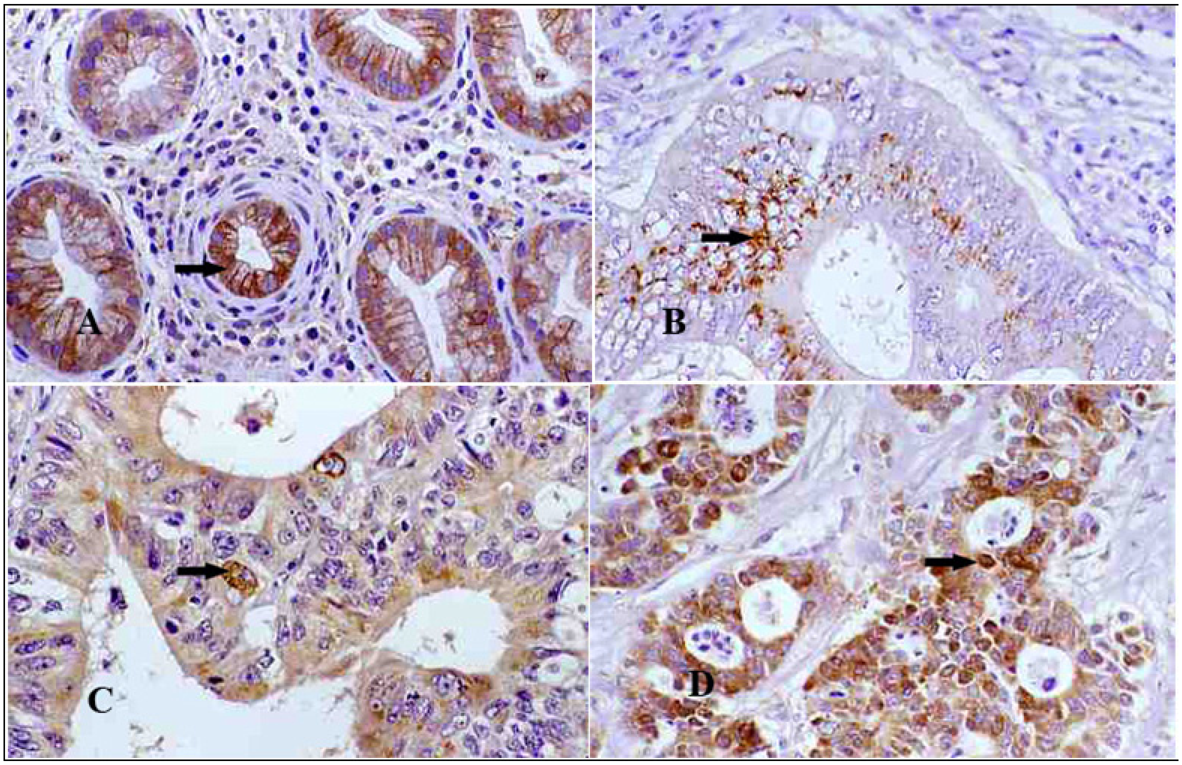

Supplementary Figure 3: KLK6, KLK7 and KLK8 immunohistochemistry in normal colonic tissues and colorectal adenocarcinoma. (A) Cytoplasmic immunohistochemical expression (IE) of KLK6 in normal colon epithelium (arrow). (B) Cytoplasmic KLK6 immunoexpression in colorectal adenocarcinoma (arrow). (C) Cytoplasmic KLK7 immunoexpression in CRC (arrow). (D) Cytoplasmic KLK10 immunoexpression in CRC (arrow). *All magnifications x200, except A x100.*

Proteomic Signatures of the Desmoplastic Invasion Front Reveal Collagen Type XII as a Marker of Myofibroblastic Differentiation During Colorectal Cancer Metastasis. Karagiannis et al

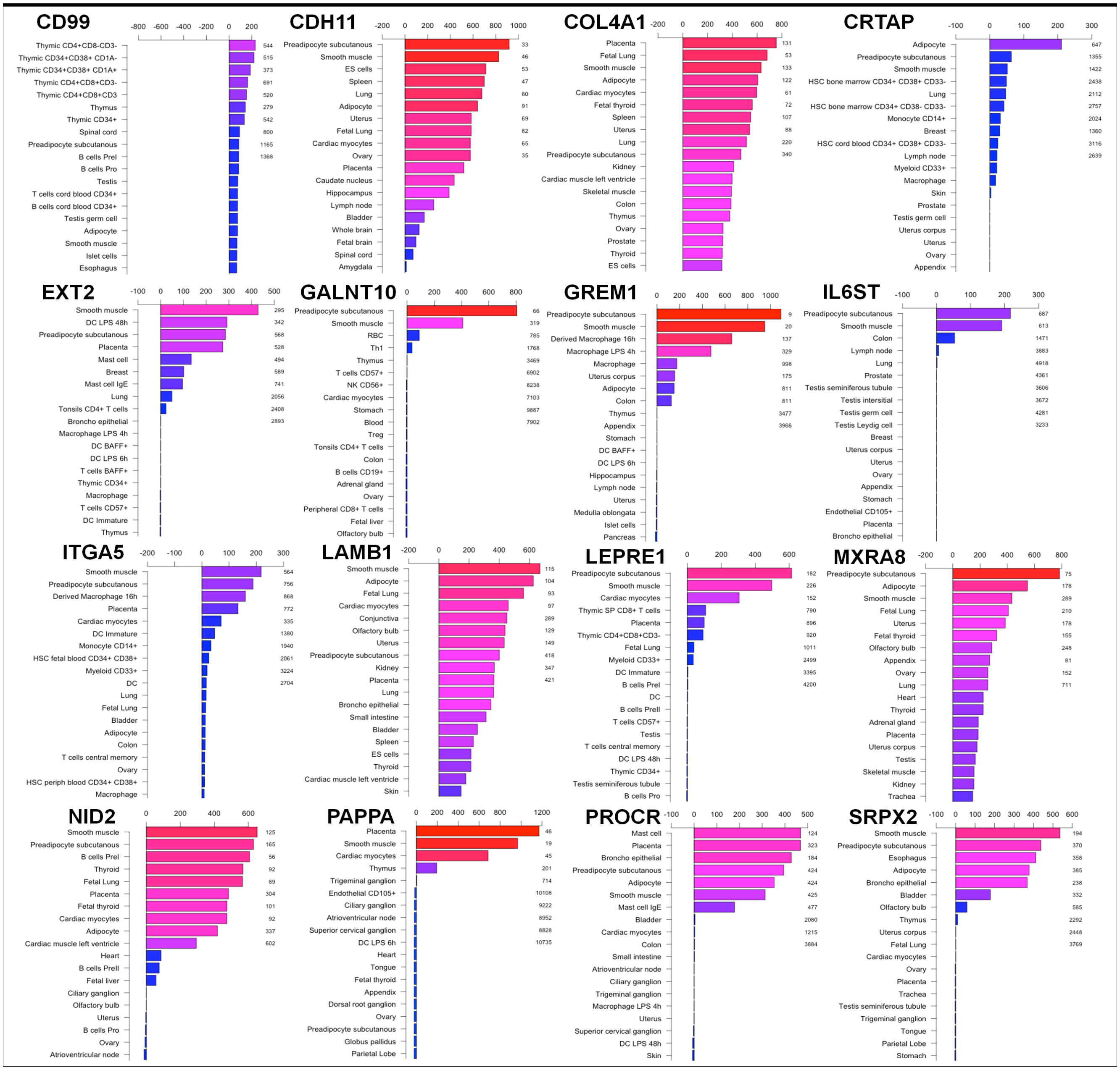

Supplementary Figure 4: Tissue specificity of sixteen of the probes from the myofibroblastic signature, using Gene Expression Profiler. The rest members of the signature were not found on the database. All the tested probes depicted "smooth muscle" among the ten highest-expressed tissues. The rows depict the gene expression levels in various tissue and cell types.

Proteomic Signatures of the Desmoplastic Invasion Front Reveal Collagen Type XII as a Marker of Myofibroblastic Differentiation During Colorectal Cancer Metastasis. Karagiannis et al

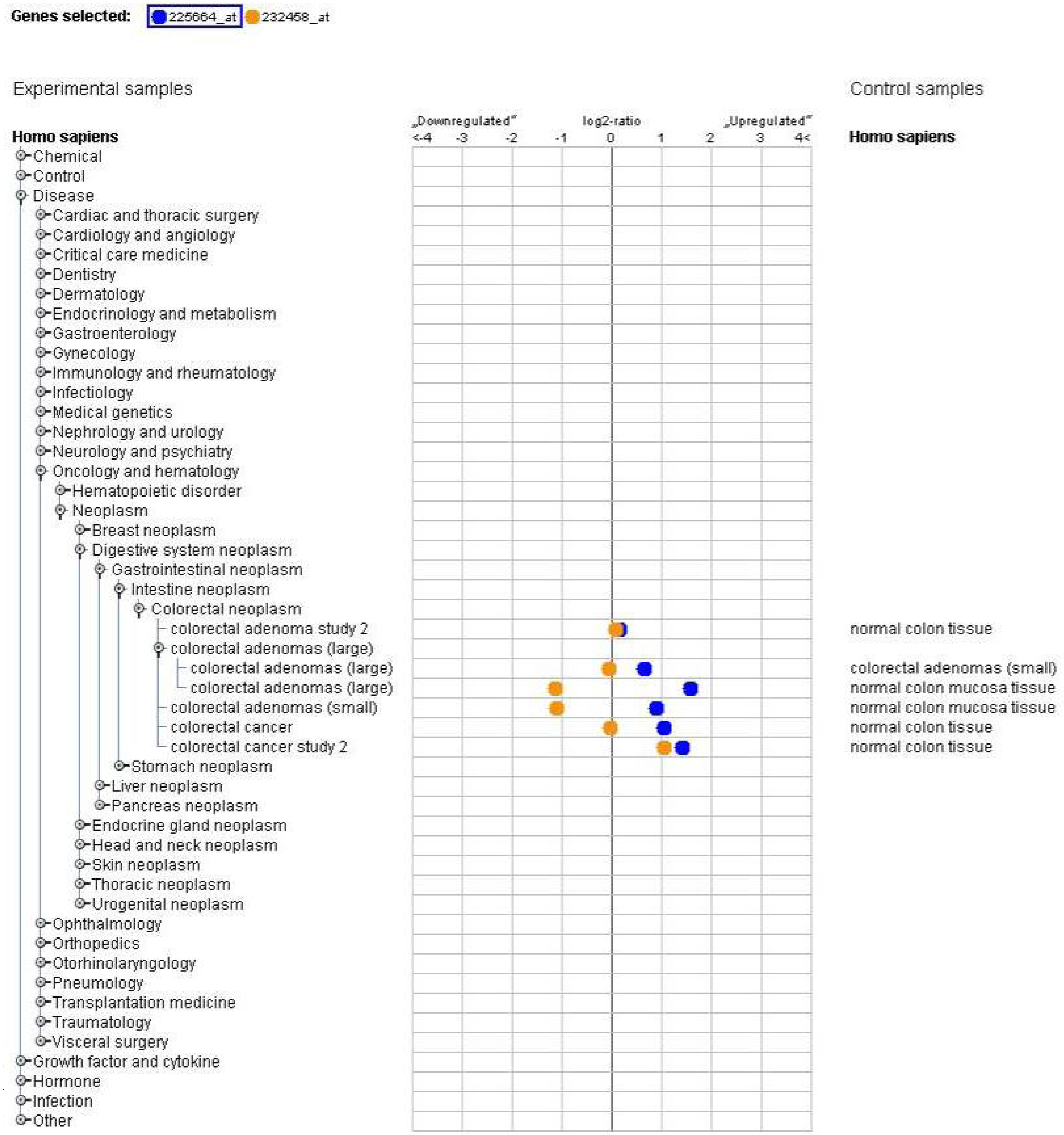

Supplementary Figure 5: Gene expression meta-analysis studies involving Collagens type III and XII, using Genevestigator. The expression ratios of the experimental conditions (left) to the control conditions (right) are depicted with Log2 ratio. As shown from the expanded tree, five experiments were used in the meta-analysis (see text for references and details).

Proteomic Signatures of the Desmoplastic Invasion Front Reveal Collagen Type XII as a Marker of Myofibroblastic Differentiation During Colorectal Cancer Metastasis. Karagiannis et al

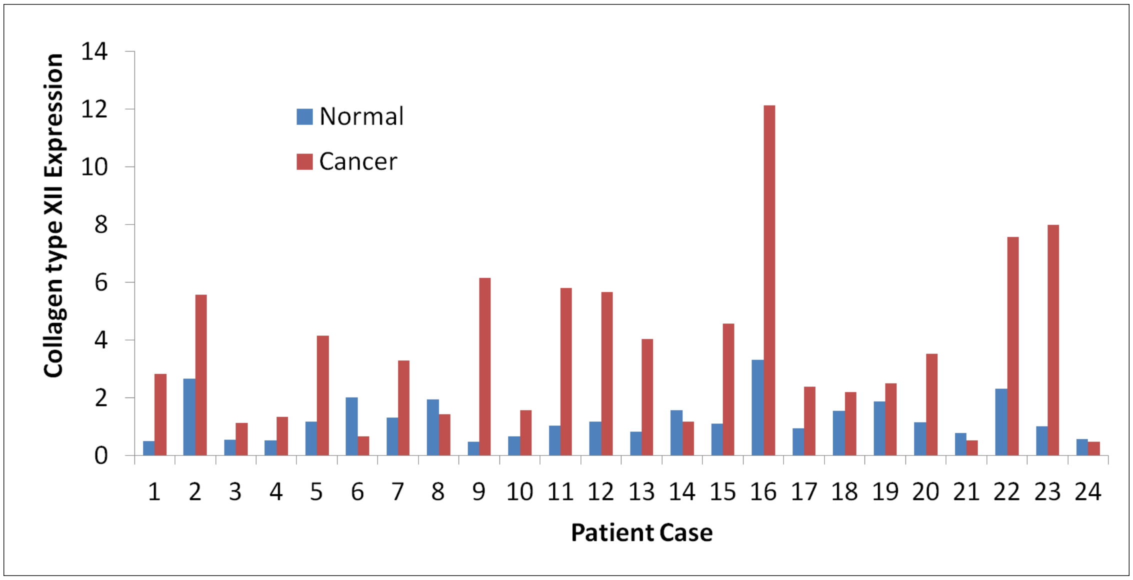

Supplementary Figure 6: COL12A1 expression in 24 CRC patients of cancerous lesions and adjacent to the tumor normal colonic mucosa, using quantitative PCR.
